# Effect of Nutrition in the First Two Years of Life on Body Composition of Preschool-Aged Children

**DOI:** 10.7759/cureus.75115

**Published:** 2024-12-04

**Authors:** Osama M Elasheer, Asmaa Ahmed Mahmoud, Merly R Rassmy, Maher M A. Farghl, Manal Darwish, Amir Mohammad AboElgheet

**Affiliations:** 1 Pediatrics, Assiut University, Assiut, EGY; 2 Public Health, Assiut University, Assiut, EGY

**Keywords:** bioelectrical impedance analysis, bmi, first 2 years of life, nutrition, obesity

## Abstract

Background

Feeding and growth during infancy have been associated with later life body mass index and early excessive weight gain is associated with obesity later on. This study aimed to assess the effect of feeding in the first two years of life on the body composition of children at the preschool age and detect the importance of using bioelectrical impedance (BIA) analysis in identifying individuals at risk of overweight and obesity.

Methods

A cross-sectional study included 160 children. Data was collected on personal history and nutritional history in detail, body composition analysis was done by a scale measuring the amount of fat, protein, water, and minerals in the body to identify individuals at-risk of overweight and obesity.

Results

The percentage of infants with a normal BMI was significantly lower in the formula-fed group (40.9%) vs. the breastfed (71.1%) and mixed-feeding groups (50.9%). Regarding the type of first food introduced, the cereal group has a higher percentage (70.4%) of infants categorized as "normal" weight, and the dairy products have a higher percentage (31.4%) of infants categorized as obese. However, there were no significant differences in total body composition or BMI evaluation according to the timing of the first food introduced. Finally, as regards the prognostic performance of BIA, the cut-off point for body fat mass is ≥5.2, and it has a high sensitivity of 85.71% and a specificity of 89.69%.

Conclusion

Body composition is affected by the type of feeding and the type of first food introduced. Body fat mass is a good predictor for discriminating between overweight and normal BMI individuals.

## Introduction

Feeding practices and growth patterns during the early months of life are linked to future body mass index levels [1]. Rapid weight increase in the initial months is a predictor of obesity in later years [2]. Current recommendations suggest exclusive breastfeeding for the initial six months, followed by the gradual introduction of specific solid foods, which is associated with reduced fat mass in children [3]. Introducing solid foods earlier than recommended can lead to quick weight gain during infancy, potentially impacting the risk of obesity in childhood [4]. Infants who followed dietary guidelines closely, consuming high amounts of fresh fruits and vegetables, meals prepared at home, and breast milk, experienced more significant weight and skin fold thickness increases between six and 12 months compared to their peers, regardless of the type of milk consumed, when solids were introduced, and maternal influences [3]. Bioelectrical impedance (BIA) is a painless, non-invasive, and easily portable technique that may clarify how the human body is operational. Body composition (BC) assessment is commonly established as a clinical technique for evaluating and estimating disease status [5]. Strong debates are still present about the effect of timing and type of introduction of complementary food on metabolic programming and later on obesity, so this study aimed to assess the effect of feeding practices in the first two years of life on the body composition of preschool-age children and to highlight the importance of using Bioelectrical Impedance Analysis (BIA) in clinical settings for identifying individuals at risk of overweight and obesity.

## Materials and methods

Study design

This is a cross-sectional study that was carried out on preschool-aged children over one year from the 1st of January 2022 to the end of December 2022. The sample size was calculated using OpenEpi, Version 3, open source calculator-SSPropor, for proportion with the prevalence of exclusive breastfeeding of 28% [6] and a total population of 350, and a confidence limit of 5%. The calculated sample was 165. A total coverage of all eligible children yielded only 160 children (with a dropout rate of 3%).

Inclusion criteria

Children aged from three to five years old, born full terms with no history of chronic disease were included in our study.

Exclusion criteria

Preterm children and full-term children who had an inborn error of metabolism or any chronic disease, congenital malformations, previous operative history, or those who were fed cow milk or buffalo milk were excluded.

Breastfeeding is defined as exclusive breastfeeding for four months and continues until 12 months of age and beyond [7]. Early introduction of the first food is giving complementary feeding to the baby before 17 weeks and late introduction of the first food is giving complementary feeding after 26 weeks [8].

**Figure 1:**
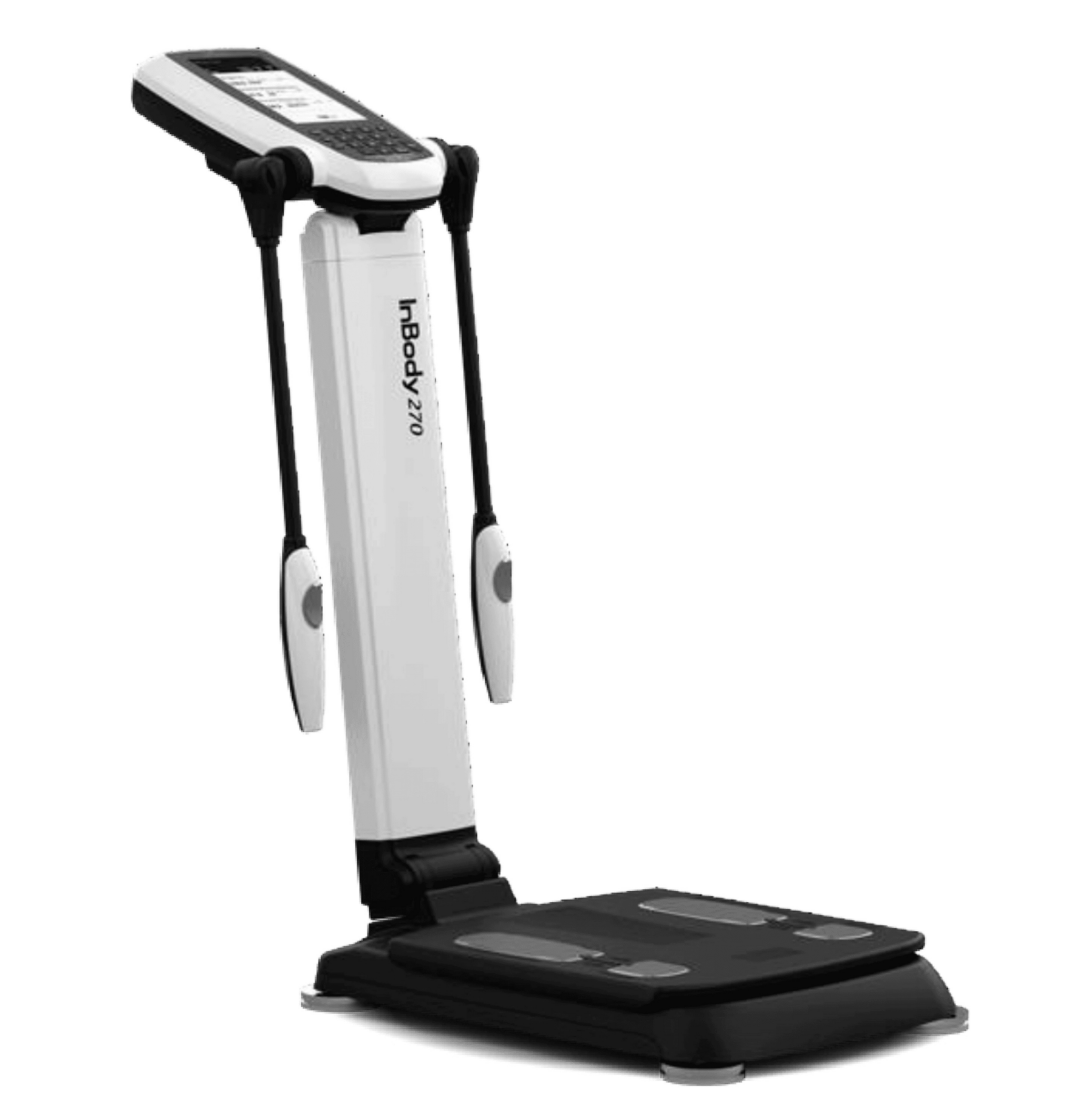
Body composition scale ACCUNIQ BC380 (ACCUNIQ, Daejeon, Republic of Korea; Image of the scale used in the nutrition unit)

Data were collected by personal interviews with mothers of eligible children in the form of personal history (name, age, sex, and weight at birth), nutritional history that includes the type of feeding (exclusive breast, formula, or mixed feeding), and type and timing of first food introduced. Body composition analysis was done by using ACCUNIQ BC380 (Figure 1).

Statistical analysis

Data were analyzed using IBM SPSS software package version 25.0 (IBM Corp., Armonk, NY, USA). Qualitative data were described using numbers and percent, using the Chi-square test as well as the Fisher's exact test for variables with small expected numbers. The Kolmogorov-Smirnov test was used to verify the normality of the distribution. Quantitative data were described using range (minimum and maximum), mean, and standard deviation, and then compared using an independent t-test or Mann-Whitney test for the analysis of data between two groups. One-way ANOVA if the comparison between more than two groups, significant level when the p-value <0.05. Receiver operating characteristic curve (ROC) was generated by plotting sensitivity (TP) on the Y axis versus 1-specificity (FP) on the X axis at different cut-off values. The area under the ROC curve denotes the diagnostic performance of the test. An area of more than 50% gives acceptable performance, and an area of about 100% is the best performance for the test.

Ethics approval and consent to participate

The study was approved by the ethics committee of the Faculty of Medicine, Assiut University (IRB no: 17100729). Written informed consents were taken from parents. Privacy and confidentiality of all obtained information were observed without intervention in the prescribed treatment.

## Results

The study included 160 full-term children; 91 of them were males (56.9%) with a mean birth weight of 3.00±0.48 kg. Breastfeeding was the most common feeding method in 83 children (51.9%) and the first food introduced to the children was vegetables and fruits in 98 children (61.3%) followed by yogurt in 35 children (21.9%). Regarding the time of the first food introduced, 42 children i.e. more than a quarter of children (26.25%) began complementary feeding early (before 17 weeks) while the majority of them (96 children, 60%) received complementary feeding at the optimum time. The average body composition for the subjects included 9.57 ± 1.83 kg of water, 2.54 ± 0.48 kg of protein, 0.88 ± 0.22 kg of minerals, 4.98 ± 2.07 kg of fat, and 5.67 ± 1.48 kg for skeletal muscle mass. The mean height, weight, and BMI of children were 105.23 ± 7.2 cm, 17.97 ± 3.50 kg, and 16.56 ± 1.79, respectively with 39.4% of children being overweight/obese (Table 1).

**Table 1:** Nutritional history and body composition analysis of studied children

	Frequency (n=160)
Feeding type	
Breastfeeding	83 (51.9%)
Formula feeding	22 (13.8%)
Mixed	55 (34.4%)
First food introduced	
Yogurt	35 (21.9%)
Vegetables / Fruits	98 (61.3%)
Fortified cereals	27 (16.9%)
Time of first food introduced	
Early >17 weeks	42 (26.25%)
Optimum (17-26 weeks)	96 (60%)
Late <26 weeks	22 (13.75%)
Body composition (mean ± SD)	
Total body water (L)	9.57 ± 1.83
Protein (kg)	2.54 ± 0.48
Mineral (kg)	0.88 ± 0.22
Body fat mass (kg)	4.98 ± 2.07
Skeletal muscle mass (kg)	5.67 ± 1.48
Height for age (cm)	105.23 ± 7.2
Weight for age (kg)	17.97 ± 3.50
BMI (Mean ± SD)	16.56 ± 1.79
BMI categories	
Normal (18.5-24.9)	96 (60.0%)
Underweight (>18.5)	1 (0.6%)
Overweight (25-29.9)	33 (20.6%)
Obese (≥30)	30 (18.8%)

Regarding factors affecting body composition, formula-fed children showed significantly higher body fat mass and mean BMI (p-values of 0.002 and 0.001, respectively). Neither the type nor timing of the first food introduced showed a significant difference in the body composition of children. As regards BMI classification, the percentage of infants with a normal BMI was significantly lower in the formula feeding group (40.9%) vs. in the breastfeeding (71.1%) and mixed feeding groups (50.9%). The percentage of infants who were obese or overweight was higher in the formula feeding group than in the other two groups with a significant association between being overweight/obese and formula feeding/mixed (p-value 0.003) (Table 2).

**Table 2:** Correlation between body composition analysis and type of feeding BMI: Body mass index Continuous data are presented as mean (±SD) and median (IQR), *=significant p-value <0.05

Variables Mean ± SD		Feeding						p-value
		Breastfeeding (n=83)		Formula feeding (n=22)		Mixed group (n=55)		
Total body water		9.35 ± 2.05		10.05 ± 1.88		9.70 ± 1.39		0.216
Protein		2.48 ± 0.54		2.66 ± 0.50		2.58 ± 0.36		0.212
Mineral		0.85 ± 0.24		0.94 ± 0.22		0.89 ± 0.18		0.197
Body fat mass		4.50 ± 1.66		6.15 ± 2.95		5.23 ± 2.03		0.002*
Skeletal muscle mass		5.49 ± 1.64		6.08 ± 1.53		5.79 ± 1.13		0.193
BMI (Mean ± SD)		16.13 ± 1.31		17.57 ± 2.52		16.80 ± 1.89		0.001*
		N	%	N	%	N	%	
BMI evaluation	Normal	59	71.1%	9	40.9%	28	50.9%	0.003*
	Underweight	1	1.2%	0	0.0%	0	0.0%	
	Overweight	17	20.5%	7	31.8%	9	16.4%	
	Obese	6	7.2%	6	27.3%	18	32.7%	

Also, obesity was significantly associated with dairy products as the first type of food introduced to the child (p = 0.041) (Table 3).

**Table 3:** Correlation between total body composition and type of first food introduced Continuous data are presented as mean (±SD) and median (IQR), *=significant p-value <0.05

Variables Mean ± SD		First food introduced						p-value between group
		Dairy product (n=35)		Vegetables / Fruits (n=98)		Fortified cereals (n=27)		
Total body water		9.57 ± 1.94		9.43 ± 1.90		10.03 ± 1.32		0.425
Protein		2.54 ± 0.53		2.51 ± 0.50		2.65 ± 0.34		0.534
Mineral		0.89 ± 0.23		0.86 ± 0.23		0.92 ± 0.15		0.458
Body fat mass		5.58 ± 2.50		4.87 ± 1.92		4.60 ± 1.92		0.247
Skeletal muscle mass		5.69 ± 1.58		5.56 ± 1.53		6.04 ± 1.08		0.428
BMI (Mean ± SD)		16.89 ± 2.38		16.54 ± 1.60		16.20 ± 1.52		0.238
		N	%	N	%	N	%	
BMI evaluation	Normal	19	54.3%	58	59.2%	19	70.4%	0.041*
	Underweight	1	2.9%	0	0.0%	0	0.0%	
	Overweight	4	11.4%	26	26.5%	3	11.1%	
	Obese	11	31.4%	14	14.3%	5	18.5%	

The timing of the first food introduced showed no significant effect on the body composition of children (Table 4).

**Table 4:** Correlation between total body composition and time of first food introduced Continuous data are presented as mean (±SD) and median (IQR), *=significant p-value <0.05

Variables Mean ± SD		Time of first food introduced						p-value between group
		Early group (n=42)		Optimum group (n=96)		Late group (n=22)		
Total body water		9.24 ± 1.74		9.82 ± 1.66		9.09 ± 2.51		0.343
Protein		2.45 ± 0.47		2.61 ± 0.43		2.40 ± 0.68		0.368
Mineral		0.83 ± 0.18		0.91 ± 0.20		0.80 ± 0.31		0.138
Body fat mass		4.73 ± 2.31		5.18 ± 1.92		4.60 ± 2.22		0.148
Skeletal muscle mass		5.42 ± 1.42		5.87 ± 1.33		5.30 ± 2.03		0.358
BMI (Mean ± SD)		16.57 ± 2.01		16.62 ± 1.65		16.28 ± 1.97		0.809
		N	%	N	%	N	%	
BMI evaluation	Normal	28	66.7%	53	55.2%	15	68.2%	0.604
	Underweight	0	0.0%	0	0.0%	1	4.5%	
	Overweight	7	16.7%	25	26.0%	1	4.5%	
	Obese	7	16.7%	18	18.8%	5	22.7%	

**Figure 2:**
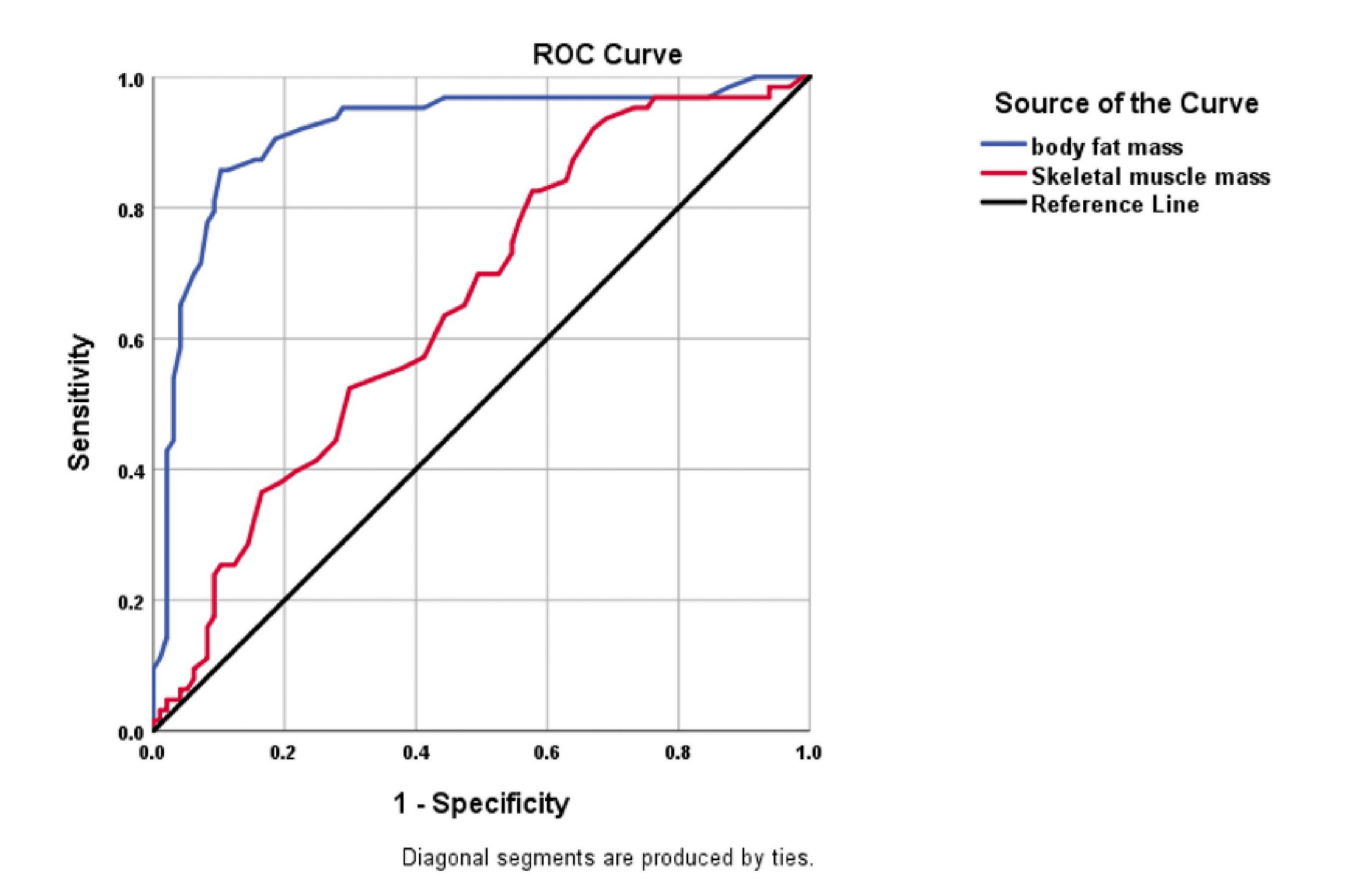
Prognostic performance of BIA to discriminate overweight patient from normal BMI

With regards to the prognostic performance of BIA to discriminate overweight patients (n = 97) from normal BMI (n = 63), it was found that BIA has significantly excellent discrimination power for fat mass (p-value < 0.001 & AUC = 0.916). The cut-off point for body fat mass is ≥5.2, and it has a high sensitivity of 85.71% and specificity of 89.69%. The positive predictive value (PPV) is 84.4%, while the negative predictive value (NPV) is 90.6%. These results suggest that body fat mass is a good predictor for discriminating between overweight and normal BMI individuals (Table 5 & Figure 2).

**Table 5:** Prognostic performance of BIA to discriminate overweight patient (n = 97) from normal BMI (n = 63) AUC: Area Under a Curve, p-value: Probability value, CI: Confidence Intervals, NPV: Negative predictive value, PPV: Positive predictive value.

	AUC	P-value	95% CI	Cut off	Sensitivity	Specificity	PPV	NPV
Fat mass	0.916	<0.001*	0.866-0.965	≥5.2	85.71	89.69	84.4	90.6
Skeletal muscle mass	0.655	0.043*	0.570-0.740	≥5.7	65.08	52.58	47.1	69.9

## Discussion

Breastfeeding has been observed to have a modest but consistent protective effect against obesity in children [9]. It is a well-established fact that traditional formula milk contains higher levels of protein compared to breast milk. Protein-rich formulas are considered a factor that can accelerate plasma insulin levels and lead to the release of insulin-like growth factor-1, consequently resulting in weight gain and later obesity [10].

Aligning with findings from the present study, Malekzadeh et al. detected that despite similar weight for age and weight-for-length Z-score (WLZ) at birth in the studied groups, WLZ was significantly higher in the formula-fed group at six months of age, indicating that formula feeding can lead to higher weight gain than breast milk in infants [11].

Li et al. found that predominantly formula-fed infants weighed more than the exclusively human milk-fed group at term-corrected age, and their greater change in weight Z-score throughout the study was accompanied by higher non-adipose tissue mass deposition [12].

Contrasting these findings, Raju et al. stated that there is no significant difference in the weight of babies who are breastfed, formula-fed, or mixed-fed [13].

When comparing the first food introduced (dairy product group, vegetable/fruit group, and fortified cereals group), the results indicated no significant differences in body composition measures between the three groups. A randomised trial studied the effect of feeding on body weight composition and insulin resistance and found that decreased weight was most associated with increased intake of legumes (r = −0.38; P < 0.0001) and decreased intake of total meat, fish, and poultry (r = +0.43; P < 0.0001). Those consuming a low-fat vegan diet also increased their intake of carbohydrates, fibre, and several micronutrients and decreased fat intake. Reduced fat intake was associated with reduced body weight (r = +0.15; P = 0.02) [14].

The results show no significant differences in body composition among the early, optimum, and late introduction of the first food groups. This suggests that the timing of the first food introduction does not significantly impact body composition in infants.

In the current study, the BMI of infants did not show significant differences based on the timing of food introduction. Similarly, a meta-analysis by Pearce et al. concluded that there is no clear association between the timing of introducing complementary foods and childhood overweight or obesity, although a very early introduction of solid foods (≤4 months of age) may result in an increase in childhood BMI [15].

A review by Qasem et al. found no difference in fat mass between groups that received complementary foods (CF) at 4 vs. 6 months of age when studied at six months of age [16].

Contrasting these findings, an earlier review by Lanigan reported an increased fat mass at follow-up in seven-year-old children who had CF introduced before 15 weeks compared to six-year-old children who had CF introduced after five months [17].

However, two systematic reviews did not find a clear association between the timing of CF introduction and measures of adiposity, such as skin-fold thicknesses, fat mass, and percentage fat mass measured by dual-energy X-ray absorptiometry and/or bioelectrical impedance [18].

In the current study, the prognostic performance of BIA to discriminate overweight children from normal BMI was evaluated; the AUC for skeletal muscle mass is 0.655, indicating that it has a fair statistically significant discrimination power (P = 0.043). The cut-off point for skeletal muscle mass is ≥5.7, and it has a sensitivity of 65.08% and specificity of 52.58%. The PPV is 47.1%, and the NPV is 69.9%. These results suggest that skeletal muscle mass is a less reliable predictor than body fat mass in discriminating between overweight and normal BMI individuals.

Aligning with this study, Khan et al. found that BIA machines vary in relative accuracy when measuring body composition in children who are obese and severely obese [18].

However, two other studies in obese/severely obese children demonstrated an underestimation of body fat percentage using an SF4 BIA device (Omron Portable Fat Analyser; Omron, Dongguan, China) or an alternate MF4 BIA (Tanita MC-180MA; Tanita, Dongguan, China) device compared to DXA, with similarly wide limits of agreement (−15 to +5%). de Silva et al., using their MF4 BIA machine-generated resistance data with both published paediatric-specific and their own derived equations for estimating body fat and free fat mass, were able to reduce systematic bias to <1%, but the limits of agreement remained wide (−9 to +9%) [19].

Limitations of the study

Only the first food introduced was asked for and not attaining a history of subsequent diets given to infants as detailed food history would be subjected to recall bias. Weight and height at the beginning of the study weren’t correlated to growth charts to determine whether it was normal for age or not. Finally, the study was conducted in an international school with a relatively high socioeconomic class compromising the variation in feeding type and practices by socioeconomic levels.

## Conclusions

Breastfeeding has a protective effect against the risk of obesity in children where formula-fed children have higher body fat mass and BMI than breastfed. A greater proportion of infants who started food with cereals had a normal BMI, in contrast to groups that started with dairy products and were mostly obese. Body fat mass as detected by BIA is a good predictor for obesity, and skeletal muscle mass is less reliable for discriminating between overweight and normal BMI individuals than body fat mass in obese.
